# Application of Cloning-Free Genome Engineering to *Escherichia coli*

**DOI:** 10.3390/microorganisms11010215

**Published:** 2023-01-15

**Authors:** Lucia Romeo, Antonia Esposito, Alberto Bernacchi, Daniele Colazzo, Alberto Vassallo, Marco Zaccaroni, Renato Fani, Sara Del Duca

**Affiliations:** 1 Department of Biology, University of Florence, 50019 Sesto Fiorentino, Italy; 2 School of Biosciences and Veterinary Medicine, University of Camerino, 62032 Camerino, Italy

**Keywords:** genetic engineering, homologous recombination, histidine biosynthesis, evolutionary mechanisms

## Abstract

The propagation of foreign DNA in *Escherichia coli* is central to molecular biology. Recent advances have dramatically expanded the ability to engineer (bacterial) cells; however, most of these techniques remain time-consuming. The aim of the present work was to explore the possibility to use the cloning-free genome editing (CFGE) approach, proposed by Döhlemann and coworkers (2016), for *E. coli* genetics, and to deepen the knowledge about the homologous recombination mechanism. The *E. coli* auxotrophic mutant strains FB182 (*hisF892*) and FB181 (*hisI903*) were transformed with the circularized wild-type *E. coli* (i) *hisF* gene and *hisF* gene fragments of decreasing length, and (ii) *hisIE* gene, respectively. His^+^ clones were selected based on their ability to grow in the absence of histidine, and their *hisF*/*hisIE* gene sequences were characterized. CFGE method allowed the recombination of wild-type *his* genes (or fragments of them) within the mutated chromosomal copy, with a different recombination frequency based on the fragment length, and the generation of clones with a variable number of in tandem *his* genes copies. Data obtained pave the way to further evolutionary studies concerning the homologous recombination mechanism and the fate of in tandem duplicated genes.

## 1. Introduction

Bacteria evolve rapidly, not only by mutation and rapid multiplication, but also by horizontal transfer of DNA, leading to strains with beneficial mutations [1]. Increasingly, studies about genes and genomes indicate that considerable horizontal transfer events have occurred in prokaryotes [2]. Besides the core genes encoding essential metabolic functions, bacterial genomes also harbor several accessory genes acquired through horizontal gene transfer (HGT) that might be beneficial under certain environmental conditions. In bacteria, HGT contributes to diversification and adaptation [3], being responsible for the widespread distribution of antibiotic resistance genes, gene clusters encoding biodegradative pathways and pathogenicity determinants, and (sub-) speciation [4]. 

Gene transfer among and within bacterial populations is predominantly mediated by conjugation, transduction, and transformation [5]. Other mechanisms involve gene transfer agents, nanotubes, membrane vesicles, and cell fusion [6,7,8,9]. The natural transformation consists of the stable uptake, integration, and functional expression of extracellular DNA that can occur under natural bacterial growth conditions; prerequisites for natural transformation include the release and persistence of extracellular DNA, the presence of competent bacterial cells, and the ability of uptaken DNA to be stabilized by integration into the bacterial genome or, in the case of plasmids, the ability to integrate or re-circularize into self-replicating plasmids [1]. Transformation is entirely directed by the recipient cell and all required proteins are encoded in the core genome [10]. For natural transformation to occur, bacterial cells must first develop a regulated physiological state of “competence”, which has been found to involve approximately 20 to 50 proteins [1]; most transformable bacteria do not permanently express these proteins but instead require specific conditions to develop competence [10]. To the extent investigated, the proportion of bacteria found to be naturally transformable is approximately 1% of the validly described bacterial species [1]. 

Bacterial transformation is a technique routinely used in laboratories for genetic engineering experiments. Although reported to occur naturally in bacteria, such as *Bacillus subtilis*, this phenomenon is generally uncommon in *Escherichia coli*, a bacterium often used in molecular biology which requires competence induction by artificial methods [11]. 

After the extracellular DNA uptake, many bacteria frequently replace existing genetic material in their genome with introgressed genes, or fragments of them, through a mechanism called “homologous recombination” [5]. Most detectable homologous recombination events in bacteria depend upon the RecA recombinase; however, a substantial level of RecA-independent recombination can be documented under some conditions. For example, RarA plays a major role in RecA-independent recombination in *E. coli*, especially for intermolecular recombination events involving short (<200 bp) homologous sequences. The role of RarA in recombination is largely obscured when RecA function is present, although its activity is enough to make a significant contribution to in vivo cloning protocols [12]. Homologous genetic recombination is essential to all organisms, for the generation of genetic diversity, the maintenance of genomic integrity, and the proper segregation of chromosomes [13]. Moreover, homologous recombination is one of the major forces driving the evolution of bacterial populations, enabling bacteria to acquire new genetic traits and adapt to changing environmental conditions [5,10]. 

The propagation of foreign DNA in *E. coli* is central to molecular biology [14]. A wide variety of approaches for targeted gene editing, involving homologous recombination, are available for *E. coli* [15]. Most of the tools for homologous recombination are based on cloning techniques (i) by using counterselection markers [16], (ii) by improving the frequency of homologous recombination using phage-derived recombinases as in ET cloning [14] and in the Datsenko and Wanner [17] method based on phage λ Red recombinase, or (iii) by realizing precise genome modifications by CRISPR-Cas9 system [15]. Other methods are based on the use of hyper-recombinogenic strains obtained by replacing bacterial recombinase genes with phage-derived recombination functions [18]. 

Recent advances have dramatically expanded the ability to engineer cells [19]. However, despite the augmented efficiency, these techniques remain time-consuming, given the need to pass through the cloning stage. To expedite genome engineering, Döhlemann and coworkers [20] developed a method for cloning-free genome editing (CFGE) in *Sinorhizobium meliloti*. The suggested technique requires fragments with phosphate at 5′ and blunt ends. Subsequently, purified DNA is circularized via self-ligation. Finally, electrocompetent cells are transformed with the purified ligation mix and transformants are selected for the resistance carried on the fragment of interest. They used this method for rapid gene inactivation in *S. meliloti*, *Agrobacterium tumefaciens* and *Xanthomonas campestris*.

The aim of the present work was to explore the possibility of using this genome editing approach for *E. coli* genetic engineering, and to deepen the knowledge about the homologous recombination mechanism. The *E. coli* mutant strain FB182 (*hisF892*), transformed with the circularized *E. coli hisF* gene, was chosen as a model for the present study. This choice relies on recent data from Del Duca et al. [21], who analyzed the frequency and type of *E. coli* FB182 natural reverse mutations under selective pressure conditions (i.e., absence of histidine in the culture medium). Data obtained revealed that only a very low percentage of the HisF^+^ revertants restored the wild-type genotype (3.8%). These data make this strain an excellent choice, thanks to the possibility of distinguishing between chromosomal revertants (harboring a different *hisF* sequence compared to the wild-type) and recombinant colonies (harboring a wild-type *hisF*), after the transformation with the *E. coli hisF* gene and plating on selective medium (i.e., in the absence of histidine).

Lastly, we applied the very same procedure to another *E. coli* histidine auxotrophic mutant, i.e., FB181 (*hisI903*), to check whether the procedure successfully applied to *E. coli* FB182 might have been restricted to this strain or suitable for any *E. coli* strain.

## 2. Materials and Methods

### 2.1. Bacterial Strains and Culture Conditions

The *E. coli* strains FB8 (wild-type *E. coli* K-12 UTH1038) [22], FB182 (*hisF892*) [23], and FB181 (*hisI903*) [23] were used in this work. *E. coli* FB182 carries a single nucleotide deletion in position 719 of the *hisF* gene, causing a frameshift and the formation of a stop codon resulting in a shorter (243 aa vs. 258 aa of the wild-type *E. coli* HisF protein) and non-functioning enzyme [24]. *E. coli* FB181 carries a single nucleotide substitution in position 311 of the *hisIE* gene, causing an amino acid substitution in the encoded protein (Phe vs. Cys of the wild-type *E. coli* HisIE protein) and the formation of a non-functioning enzyme (this work). 

The cloning strategies were carried out with E. coli DH5α (F–endA1 glnV44 thi1 recA1 relA1 gyrA96 deoR nupG Φ80dlacZΔM15 Δ(lacZYA-argF)U169 hsdR17(rK–mK+) λ-) (laboratory stock).

Cells were grown in LB medium [25], supplemented with agar 1.6% *w/v*, ampicillin 100 μg/mL, X-Gal 40 μg/mL, and IPTG 50 μg/mL when required. 

*E. coli* FB182 CFGE and complementation assays were performed on minimal medium Davis (MMD) [26] ((NH_4_)_2_SO_4_ 1 g/L; K_2_HPO_4_ 7 g/L; KH_2_PO_4_ 2 g/L; Na_3_-citrate·2H_2_O 0.5 g/L; MgSO_4_·7H_2_O 0.1 g/L; pH 7.2) with agar 1.6% *w/v*, glucose 1% *w/v*, and histidine 25 μg/mL when required. All strains were cultivated at 37 °C. 

### 2.2. Plasmids, Genes, and Cloning Procedures 

**Figure 1:**
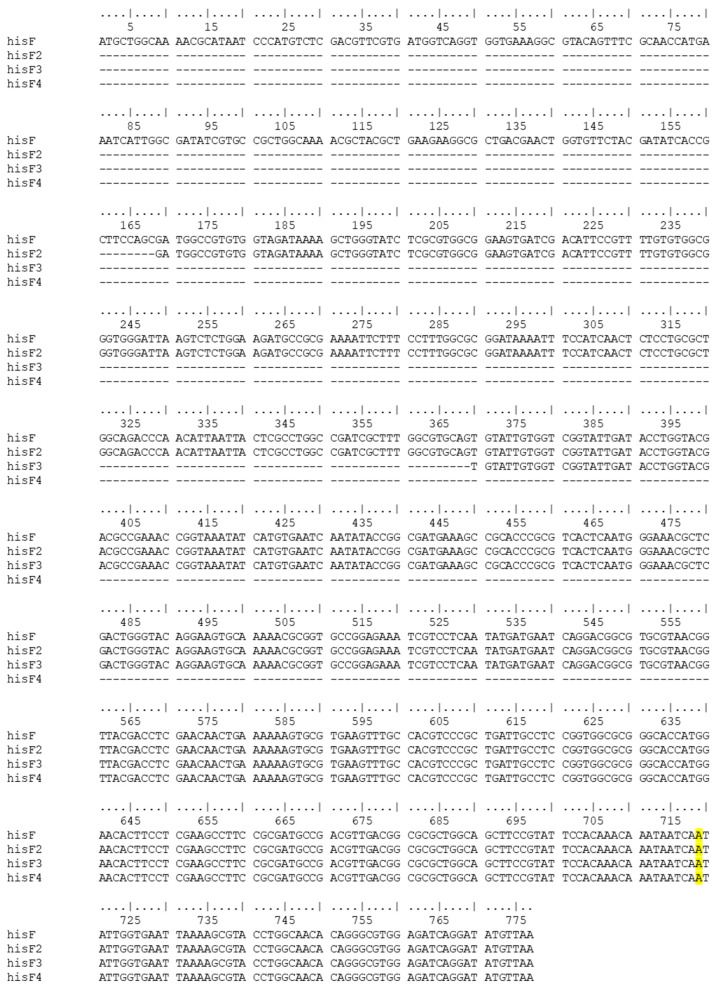
Nucleotide sequences of the *hisF* (777 bp), *hisF2* (609 bp), *hisF3* (408 bp), and *hisF4* (217 bp) fragments. The nucleotide which is deleted in *E. coli* FB182 (*hisF892*) is highlighted in yellow.

The entire *E. coli* wild-type *hisF* gene (NCBI ID: 946516) (777 bp) and fragments of the wild-type *hisF* gene (*hisF2*: 609 bp, *hisF3*: 408 bp and *hisF4*: 217 bp) (Figure 1) that overlap the *E. coli* FB182 *hisF* single nucleotide deletion were cloned into pGEM-T Easy vector (Promega) through TA cloning. 

The same procedure was used for the entire *E. coli* wild-type *hisIE* gene (NCBI ID: 946515) (869 bp). 

The amplification of the *hisF*, *hisF2*, *hisF3*, and *hisF4* fragments was performed in a 20 μL reaction volume with 0.2 μM of primers coli_hisF FW, coli_hisF_2 FW, coli_hisF_3 FW, or coli_hisF_4 FW, respectively, and coli_hisF REV (Table 1), 0.4 U of Phusion High-Fidelity DNA Polymerase (ThermoFisher Scientific, Waltham, MA, USA), and 2 μL of *E. coli* FB8 thermal lysate as template. The PCR cycling was set up using an annealing temperature of 56 °C. The amplification of the *hisIE* gene was performed in a 20 μL reaction volume with 0.2 μM of primers coli_hisIE_ext FW and coli_hisIE_ext REV (Table 1), 0.4 U of Phusion High-Fidelity DNA Polymerase (ThermoFisher Scientific, Waltham, MA, USA), and 2 μL of *E. coli* FB8 thermal lysate as template (annealing temperature of 60 °C). 

Amplicons were visualized through a 0.8% *w/v* agarose gel electrophoresis and purified. The addition of A-overhangs was performed through incubation at 72 °C for 30 min in the presence of 1 U of DreamTaq Polymerase (ThermoFisher Scientific, Waltham, MA, USA), before proceeding with the TA cloning in the pGEM-T Easy vector. *E. coli* DH5α chemically competent cells were used for all cloning procedures. Plasmid extraction was performed using QIAprep Spin Miniprep Kit (Qiagen, Hilden, Germany). 

### 2.3. PCR and Sanger Sequencing

The pGEM-T Easy inserts were amplified using 0.05 μM of M13 FW and M13 REV primers (Table 1), 1 U of DreamTaq DNA Polymerase (ThermoFisher Scientific, Waltham, MA, USA), and 1 μL of cell thermal lysate as template, with an annealing temperature of 56 °C. 

The amplification of *E. coli* FB182 *hisF* gene, after the CFGE experiment, was performed using 0.2 μM of primers coli_hisF_ext FW and coli_hisF_ext REV (Table 1), 1 U of DreamTaq DNA Polymerase (ThermoFisher Scientific, Waltham, MA, USA), and 1 μL of cell thermal lysate as template (annealing temperature of 59 °C). The amplification of *E. coli* FB181 *hisIE* gene, after the CFGE experiment, was performed using 0.2 μM of primers coli_hisIE_ext FW and coli_hisIE_ext REV (Table 1), 1 U of DreamTaq DNA Polymerase (ThermoFisher Scientific, Waltham, MA, USA), and 1 μL of cell thermal lysate as template (annealing temperature of 60 °C). 

PCR products were purified using ExoSAP-IT™ Express PCR Product Cleanup (Applied Biosystems, Waltham, MA, USA). The sequencing reaction was performed in a 10 μL volume with 1 μL BigDye™ Terminator v3.1 Ready Reaction Mix (Applied Biosystems, Waltham, MA, USA), 0.32 μM of primer forward or reverse, and 1 μL of purified PCR product as template. Sequencing reactions were purified using BigDye Xterminator™ (Applied Biosystems, Waltham, MA, USA), and capillary electrophoresis was run in a SeqStudio Genetic Analyzer (ThermoFisher Scientific, Waltham, MA, USA). Sequencing data were analyzed using BioEdit [27].

### 2.4. Cloning-Free Genome Editing

The *hisF*, *hisF2*, *hisF3*, *hisF4*, and *hisIE* fragments were extracted from the pGEM-T Easy vector using *Eco*RI enzyme, which cuts on both sides of the cloning site. Then, they were purified from 0.8% *w/v* agarose gel with QIAquick Gel Extraction Kit (Qiagen, Hilden, Germany), and finally circularized by adding 5 U of T4 ligase (ThermoFisher Scientific, Waltham, MA, USA). Self-ligation was performed at 14 °C overnight. Based on the work of Döhlemann et al. [20], in which electrocompetent cells were transformed with 450 ng of 1500 bp purified ligation products, 250 ng, 200 ng, 130 ng, 70 ng, and 280 ng of *hisF*, *hisF2*, *hisF3*, *hisF4*, and *hisIE* fragments were circularized through ligation, respectively. Ligation products were ethanol-precipitated and resuspended in 5 μL of dH_2_O. *E. coli* FB182 and *E. coli* FB181 electrocompetent cells were transformed through electroporation with the 5 μL purified ligation reaction. After the electric shock, bacterial cells were resuspended in 1 mL of SOC medium and incubated for 1.5 h at 150 rpm at 37 °C. Then, cell suspensions were washed twice in saline solution (NaCl 0.9% *w/v*), 10^−4^ and 10^−6^ dilutions were plated on LB Agar to calculate the vital titer, and the total remaining cell suspension as plated on MMD with glucose 1% *w/v*. An aliquot of electrocompetent cells was used as a negative control, electroporated but not transformed. MMD plates were incubated at 37 °C for 72 h.

### 2.5. Genomic DNA Extraction and MinION Nanopore Sequencing

To obtain the genomic DNA (gDNA) of *E. coli* strains, single colonies were inoculated in 10 mL of LB and incubated at 37 °C overnight under shaking. Genomic DNA was extracted using PowerLyzer PowerSoil DNA Isolation Kit (MO BIO Laboratories, Carlsbad, CA USA), following the manufacturer’s instructions. gDNA was visualized through a 0.8% *w/v* agarose gel electrophoresis and quantified using a Qubit 4 Fluorometer and Qubit dsDNA HS Kit (Invitrogen, Waltham, MA, USA).

Nanopore sequencing was performed with a PCR-free approach following the native barcoding genomic DNA protocol provided by Oxford Nanopore Technologies (ONT) (v. NBE_9065_v109_revY_14Aug2019), as reported in Semenzato et al. [28]. An amount of 1 μg of each input gDNA was repaired and end-prepped using the NEBNext Companion Module for Oxford Nanopore Technologies Ligation Sequencing (New England Biolabs, Ipswich, MA, USA). Upon purification with Agencourt AMPure XP beads (Beckman Coulter, Brea, CA, USA) on a magnetic separator, concentrations of DNA samples were determined using Qubit. Then, 500 ng of each end-prepped DNA sample were barcoded using Native Barcoding Expansion 13–24 (ONT) and NEB Blunt/TA Ligase Master Mix (New England Biolabs, Ipswich, MA, USA). After a purification step, equimolar amounts of barcoded DNA samples were pooled to have a total of 700 ng and were subjected to the adapter ligation. DNA library was enriched with >3 kb long fragments during the subsequent clean-up step using the Long Fragment Buffer included in the Ligation Sequencing Kit (ONT). DNA library was immediately sequenced; therefore, an R9.4.1 Flow Cell (ONT) was primed with the Flow Cell Priming Kit (ONT). The library was loaded following the instruction provided by the protocol and sequencing was performed with a MinION MK1B (ONT) using the MinKNOW software (v. 21.10.4) for 72 h. Base-calling in high accuracy mode and demultiplexing were performed using Guppy (v. 4.3.4).

### 2.6. Genome Assembly, Annotation, and Analyses

The quality of the obtained reads was evaluated by inspecting them with FastQC software (v. 0.73) [29]. De novo assembly was performed using Canu assembler software (v.2.1.1) [30] and the quality of contigs was evaluated by QUAST (v.5.0.2) [31]. Local BLASTn of the *his* genes on the obtained contigs was performed [32,33]. All these procedures were performed in a Galaxy environment (Galaxy Version 2.10.1 + galaxy0). 

Differences among the average numbers of the obtained transformants were evaluated through an analysis of variance (ANOVA) using Tukey’s pairwise test.

### 2.7. Prediction of Protein Three-Dimensional Structure

The three-dimensional structure of HisIE protein encoded by *E. coli* FB181 was predicted with RoseTTAFold software [34] through a comparative modeling approach. The template used was *E. coli* K12 HisIE, predicted through AlphaFold2 [35] and available on the AlphaFold2 Protein Structure Database (accession number P06989). Three-dimensional structures were superposed using UCSF Chimera (v. 1.16) [36].

### 2.8. Isolation of HisI^+^ Revertants

*E. coli* FB181 HisI^+^ revertants were obtained as follows:

- *E. coli* FB181 cells were grown overnight at 37 °C with shaking (150 rpm) in minimal medium Davis (MMD) [26] with glucose 1% and histidine 25 μg/mL.
- The optical density (O.D.600) of the culture was measured and the culture was diluted to O.D.600 0.1 in a final volume of 50 mL of MMD containing glucose 1% and histidine 25 μg/mL.
- The culture was then incubated at 37 °C with shaking (150 rpm). At the end of the log phase, cells were centrifuged, washed twice in saline solution (NaCl 0.9% *w/v*), and then spread on 100 mL MMD plates containing agar 1.6% and glucose 1% in the absence of histidine (three plates), or in the presence of histidine 0.3 μg/mL (three plates) or 1 μg/mL (three plates). An amount of 100 μL of 10^−5^ and 10^−6^ dilutions were plated on LB agar [25] to evaluate the cells’ vital titer.
- Vital titer plates were incubated at 37 °C overnight. Selective pressure plates were incubated at 37 °C for 15 days, and the appearance of HisI^+^ revertants was checked daily.
- HisI^+^ revertants were tested for their ability to grow in the absence or in low concentrations of histidine through streaking on MMD plates containing glucose 1% and histidine 0, 0.3, 1 μg/mL.

## 3. Results

### 3.1. CFGE of E. coli Wild-Type hisF Gene in E. coli FB182

To test whether the CFGE approach was applicable to *E. coli*, the transformation of *E. coli* FB182 (*hisF892*) with *E. coli* wild-type *hisF* gene was performed. As a preliminary test aiming to optimize the procedure (i.e., to avoid the possibility that some incorrect nucleotides might be incorporated during the PCR amplification), the gene was cloned into pGEM-T Easy vector and used for *E. coli* DH5α transformation. The correctness of the insert was checked through Sanger sequencing. 

Then, the *hisF* gene was cleaved from the recombinant plasmid using *Eco*RI, purified, circularized via self-ligation, and used for *E. coli* FB182 transformation through electroporation. After transformation, cells were plated on minimal medium (MMD) with 1% glucose in the absence of histidine, and incubated at 37 °C for 72 h. The experiment was performed twice. 

The vital titer of transformed cells ranged between 10^6^ and 10^7^ CFU/mL. To check the possibility that *E. coli* FB182 His^+^ colonies grown on MMD owned a non-cut recombinant plasmid (i.e., pGEM-T Easy–*hisF*), enabling cell growth on minimal medium lacking histidine, colonies were streaked on LB in absence and in presence of ampicillin. Clones able to grow in the presence of the antibiotic were discarded (about 13% for each experiment). 

The number of revertants obtained in the two experiments is shown in Table 2. 

No colonies grew on the MMD plate containing the non-transformed negative control *E. coli* FB182. Then, twenty colonies were randomly chosen from both experiments, and their ability to grow in the absence of histidine was furtherly confirmed by streaking them on MMD plates with 1% glucose. To evaluate if the mutated chromosomal *hisF* gene was replaced with the wild-type *hisF* gene acquired by the transformation in His^+^ transformant clones, the *hisF* region was amplified from the 40 selected revertants using the coli_hisF_ext FW and coli_hisF_ext REV primers (which anneal, externally of *hisF*, inside the *hisA* and *hisIE* genes, respectively). 

**Figure 2:**
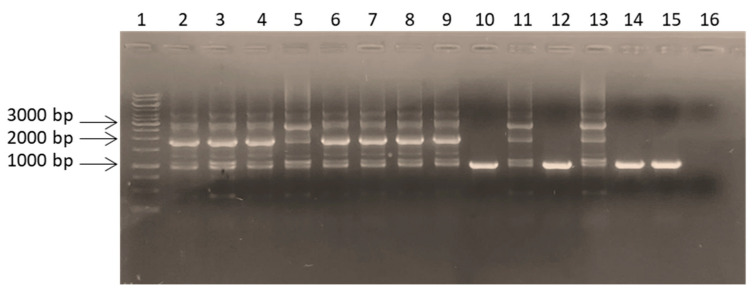
PCR amplicons, obtained using coli_hisF_ext FW and coli_hisF_ext REV primers, of some His^+^ revertants obtained from the CFGE experiments. Lanes: (1) GeneRuler 1 kb DNA ladder (ThermoFisher Scientific, Waltham, MA, USA); (2–15) *hisF* amplicons from fourteen HisF^+^ revertants; (16) PCR negative control.

**Figure 3:**
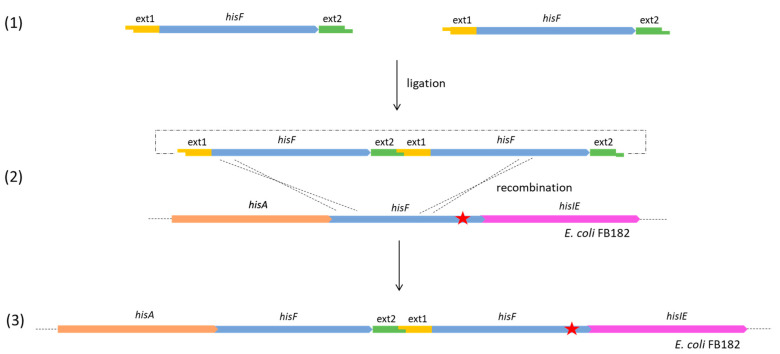
A possible molecular rearrangement leading to a His^+^ clone harboring two in tandem *hisF* and retaining the mutated copy. The red star corresponds to the *E. coli* FB182 *hisF892* single nucleotide deletion.

An example of data obtained is shown in Figure 2, of which analysis revealed that some clones harbored an amplicon of the expected size (959 bp). In contrast, others exhibited an amplicon larger than the size of the expected one, suggesting that one, two, or three copies might have been integrated into the host chromosome, possibly (at least partially) replacing the *hisF*-mutated gene. However, as an alternative scenario, it is possible that in His^+^ revertants harboring more copies of the *hisF* genes, the mutated one might have been retained, and that the wild-type copies might have recombined within the *hisF*-mutated sequence outside the mutation site, giving rise to a *hisF* region double the size of the residing one (Figure 3).

PCR products showed a pattern of bands with different lengths, likely due to PCR artifacts caused by the redundancy of *hisF* copies, with a more intense band corresponding to double or triple the size of the single-copy band (Figure 2). In order to check this hypothesis, the nucleotide sequence of each amplicon was Sanger sequenced, and the data obtained are summarized as follows:

- Seven revertants owned a wild-type *hisF* gene replacing the *E. coli* FB182 mutated one (group A). Based on the previous assumption (i.e., the very low probability, less than 4%, of spontaneous restoring of the correct sequence) [21], they should be the result of a recombination event involving a single copy of the wild-type *hisF* gene;
- Thirty-two revertants possessed two or more in tandem *hisF* copies (group B) and are the result of recombinational events involving one, two, or more copies of the donor DNA;
- Just one transformant was a chromosomal revertant owning a *hisF* gene with a restored frame but a different sequence from the wild-type one (group C), according to Del Duca et al. [21].

In order to confirm the presence of single, double, or triple copies of the *hisF* gene in different HisF^+^ revertants and to avoid the possibility that other *hisF* wild-type copies might have been integrated into different chromosomal *loci*, the genomic DNA was extracted from *E. coli* FB182 and four representatives of group B (40_E1 and 50_E2, having presumably two in tandem *hisF* copies, and 55_E1 and 20_E2, harboring presumably three in tandem copies) and sequenced through MinION Nanopore technology, as described in Materials and Methods. Metrics of the obtained assemblies are reported in Table 3.

Since the obtained largest contigs presumably covered the majority of the *E. coli* chromosome, a local BLASTn of all *his* genes was directly performed on the assembly outputs, obtaining the following results:

- In *E. coli* FB182, only one copy of each *his* genes was found, located inside the compact *E. coli his* operon, and harboring, as expected, the single nucleotide deletion in position 719 of *hisF*.
- For the clones belonging to group B, the complete genome analysis confirmed the presence of two in tandem *hisF* copies for 40_E1 and 50_E2, and three in tandem copies for 55_E1 and 20_E2. The analysis of the sequences allowed us to verify that, in all four clones, the last *hisF* copy carries the *E. coli* FB182 single nucleotide deletion, while the other one/two copies correspond to wild-type *hisF*. Moreover, the region between the in tandem *hisF* copies consists of the pGEM-T Easy vector region comprised between the *Eco*RI restriction sites and the TA cloning insertion site. This finding can be explained as follows: during the ligation step, two or more *hisF* copies joined each other with their *Eco*RI overhanging ends and then recombined with the *E. coli* FB182 *hisF* gene, as shown in Figure 3.
- No additional *hisF* gene or part thereof was found outside the *his* genomic *locus* in any of the four HisF^+^ revertants analyzed.

The clones in which recombination allowed the substitution of the mutated *E. coli* FB182 *hisF* gene with the wild-type gene (group A) resulted in 7 out of 40 characterized colonies. The total His^+^ clones obtained in the two experiments were 121; by assuming that the randomly chosen 40 investigated colonies were representative of the distribution of the molecular rearrangements that occurred in the two experiments, the number of recombinants belonging to group A would be approximately 21. Based on the obtained vital titer of transformed cells (10^6^–10^7^ CFU/mL), the frequency of recombination leading to the correct substitution of the mutated *E. coli* FB182 *hisF* gene with the wild-type gene (group A) would be around 10^−6^. 

### 3.2. CFGE of E. coli Wild-Type hisF Gene Fragments in E. coli FB182

The possibility of using shorter wild-type *hisF* fragments, comprising the position 719 of *hisF* (i.e., the site of the single nucleotide deletion in *E. coli* FB182), to allow *hisF* gene recombination in *E. coli* FB182, was investigated. 

Three *hisF* gene fragments (i.e., *hisF2*, *hisF3*, and *hisF4* with a size of 609, 408, and 217 bp, respectively) were amplified from the wild-type *E. coli* strain FB8 DNA and cloned into the pGEM-T Easy vector. The correctness of the inserts was checked through Sanger sequencing. Then, the genes were cleaved from the recombinant plasmids using *Eco*RI, purified, circularized via self-ligation, and used for *E. coli* FB182 transformation through electroporation. After transformation, cells were plated on minimal medium MMD with 1% glucose, and incubated at 37 °C for 72 h. This experiment was performed in triplicate, and the replicates were named 3, 4, and 5, respectively. 

The obtained vital titers were in the order of 10^6^ CFU/mL. *E. coli* FB182 His^+^ colonies grown on MMD medium after 72 h incubation were streaked on LB with ampicillin to discard those carrying the recombinant plasmid. The number of the remaining transformant colonies is reported in Table 2. 

Only one colony, among the three replicates, grew on an MMD plate containing the non-transformed negative control *E. coli* FB182. A total of 20 His^+^ colonies were randomly chosen among those obtained upon the transformation with the entire wild-type *hisF* for experiments 3, 4, and 5, together with all the colonies obtained from the transformation with *hisF* fragments. Their ability to grow without histidine was confirmed by streaking them on MMD plates with 1% glucose lacking histidine. The *hisF* gene from His^+^ revertants was amplified using coli_hisF_ext FW and coli_hisF_ext REV primers, and Sanger sequenced. Data obtained are reported in Table 4.

**Figure 4:**
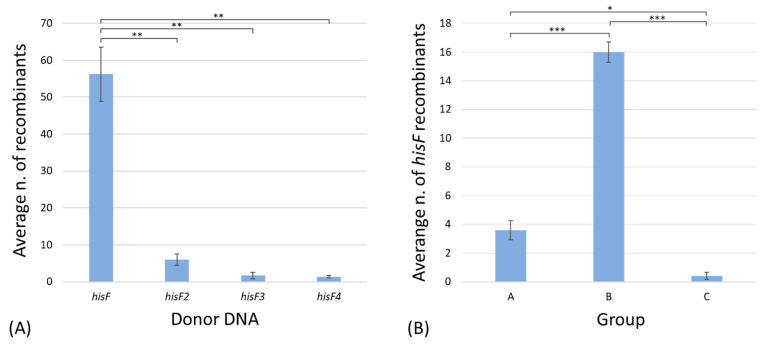
(**A**) Average numbers of His^+^ colonies obtained from the 5 experiments, divided on the basis of the length of the *hisF* fragment used for the transformation. (**B**) Average numbers of His^+^ colonies obtained from the transformation with the *hisF* gene across the 5 experiments, divided on the basis of the different groups. Bars represent standard errors. Significant differences were evaluated through analysis of variance (ANOVA) performed using Tukey’s pairwise test. Asterisks indicate significant differences (*: *p*-value < 0.01; **: *p*-value < 0.001; ***: *p*-value < 0.0001).

On the basis of the obtained data, *hisF* recombination frequency decreases with the decreasing of the fragment length (Figure 4A). 

The average number of transformant colonies was calculated for every experiment. Then, an analysis of variance was performed among the different groups, defined on the basis of the donor DNA used for transformation (Table S1). Results highlighted a significant difference between the number of His^+^ colonies obtained after the transformation with the entire *hisF* gene and the *hisF* fragments.

Moreover, the average number of transformant colonies obtained in the five experiments following the transformation with the *hisF* gene, separated on the basis of the different groups (i.e., the different molecular rearrangements) was also calculated (Figure 4B; Table S2). The differences among the three groups were all statistically significant, highlighting a preponderance of the transformants harboring two or more in tandem *hisF* copies, and a very low occurrence of chromosomal revertants (group C). However, they were considered revertants only for those clones carrying a *hisF* sequence different from that of *E. coli* wild-type *hisF*; we cannot a priori exclude that few colonies belonging to group A gained the wild genotype through reversion instead of recombination.

### 3.3. CFGE of E. coli Wild-Type hisIE Gene in E. coli FB181

In order to check whether the CFGE procedure used for the *E. coli* strain FB182 might not be restricted to the *hisF* gene case, the same procedure was applied to another histidine auxotrophic mutant (i.e., the *E. coli* strain FB181 *hisI903*) harboring a mutation in the *hisIE* gene. This gene encodes a bifunctional enzyme catalyzing the second and third steps of histidine biosynthesis [37].

**Figure 5:**
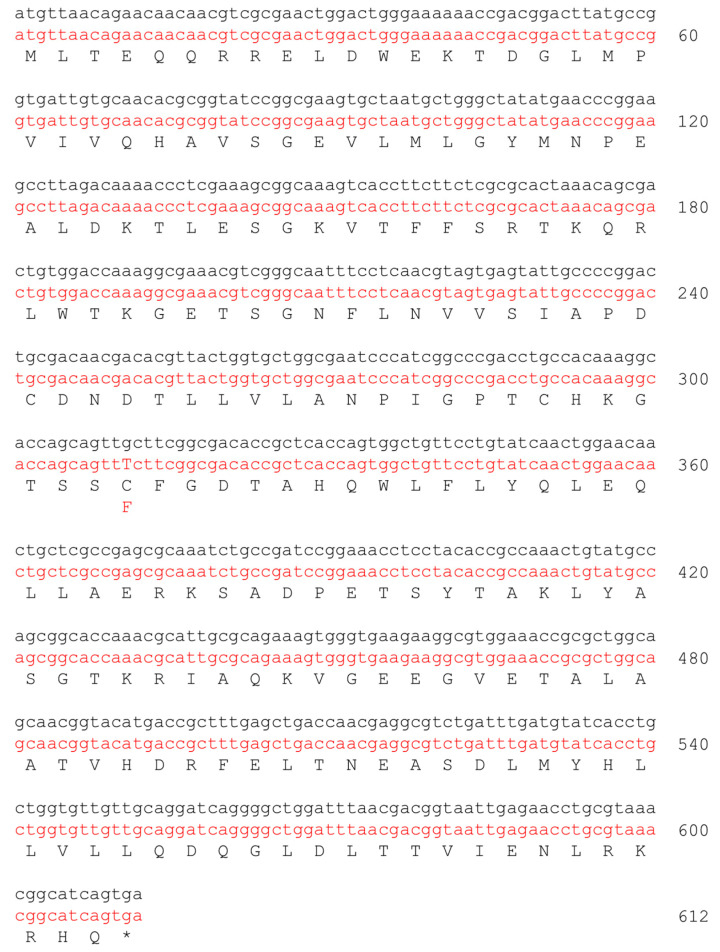
Nucleotide sequence of the *hisIE* gene from *E. coli* wild-type (black) and the mutant strain *E. coli* FB181 (red). The amino acid sequence of the encoded protein is reported in upper case. Asterisk indicates the stop codon in *E. coli hisIE* sequence.

Firstly, the nucleotide sequence of the *hisIE* gene from the mutant strain *E. coli* FB181 was determined. To this purpose, the entire *hisIE* gene was PCR-amplified using the primers coli_hisIE_ext FW and coli_hisIE_ext REV and sequenced (as described in Section 2). As shown in Figure 5, the *hisIE* gene from strain FB181 harbored a single point mutation in position 311: the transversion G vs. T modified the corresponding codon, replacing a cysteine (in position 104) with phenylalanine. 

**Figure 6:**
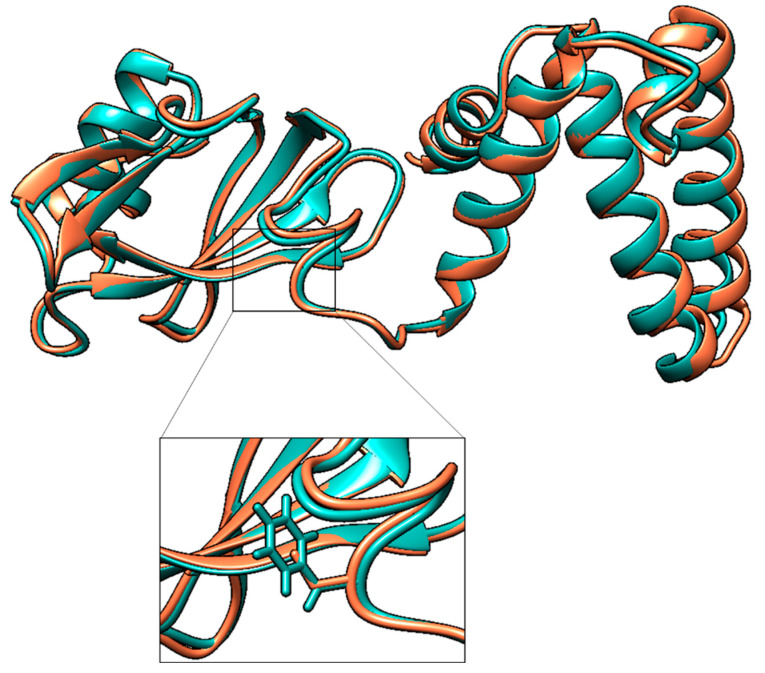
Prediction of the three-dimensional structure of *E. coli* FB181 HisIE protein (in cyan) superimposed on the three-dimensional structure of wild-type *E. coli* K12 HisIE available on the AlphaFold2 Protein Structure Database (accession number P06989) (in pink).

This mutation modifies the protein’s three-dimensional structure, affecting its catalytic activity (Figure 6). Indeed, the *E. coli* FB181 mutant strain fails to grow on MMD in absence of histidine. Moreover, as observed for *Shigella flexneri* HisIE enzyme [38] (sharing 98.5% sequence similarity with *E. coli* HisIE), the Cys104—together with Cys97 and His98—is required for the coordination of a zinc ion, essential for its catalytic activity. 

We also determined the frequency of spontaneous reversion to the His^+^ phenotype of *E. coli* FB181 cells as described in Materials and Methods. The experiment was performed in triplicate, and for each replica about 8 × 10^8^ cells were plated on MMD either in the absence or in the presence of traces of histidine (i.e., 0.3 and 1.0 µg/mL). The appearance of HisI^+^ revertants was checked daily for 15 days. Data obtained revealed that no His^+^ revertant was found in the absence of histidine in any of three replicas. Hence, the frequency of spontaneous reversion in the absence of histidine was lower than 1.6 × 10^−10^. We obtained HisI^+^ revertants only on MMD plates in the presence of 0.3 and 1.0 µg/mL of histidine (three and eight revertants, respectively), which appeared after three days of incubation at 37 °C. 

Once these data were obtained, the CFGE was performed on the *E. coli* FB181 mutant strain using the very same procedure applied to the *hisF* mutant strain.

The experiment was carried out as described in Materials and Methods and 26 His^+^ transformants were obtained on MMD in the absence of histidine after 72 h incubation. As it might be expected based on the very low frequency of spontaneous reversion of *E. coli* FB181 in the absence of histidine, no His^+^ revertants were found in the control plate (i.e., cells subjected to electroporation in the absence of DNA). Indeed, the obtained vital titer was in the order of 10^6^ CFU/mL. *E. coli* FB181 His^+^ colonies were streaked on LB with ampicillin to discard those carrying the recombinant plasmid, and none of them grew in the presence of this antibiotic.

The *hisIE* gene was then amplified from each of the 26 colonies and data obtained revealed that an amplicon with the expected size was detected in all samples (not shown). Sanger sequencing revealed that all the amplicons harbored the wild-type *hisIE* sequence, suggesting the occurrence of transformation. 

The efficiency of this technique, defined as the number of transformants *per* µg of donor DNA, was calculated for all the genes/gene fragments used in this study (Table S3). Data obtained highlighted a reduction in the efficiency of transformation with the decrease in the length of the DNA fragment. 

## 4. Discussion and Conclusions

The necessity of obtaining a molecular technique to easily perform genome editing on *E. coli* is of utmost importance. This work aimed to explore the possibility of using a CFGE approach for *E. coli* genetic engineering, and to better investigate the homologous recombination which underlies CFGE methods, together with the molecular mechanisms at the basis of genome evolution. For this purpose, the transformation of *E. coli* FB182 with *E. coli* wild-type *hisF* gene was performed. Five different experiments were carried out, demonstrating that the transformation of *E. coli* FB182 with the circularized wild-type *hisF* gene allows its recombination with the chromosomal copy, generating His^+^ colonies carrying a wild-type *hisF*. Moreover, even shorter *hisF* fragments allow *hisF* recombination; however, obtained results highlighted a lower recombination frequency for the *hisF* fragments than the entire *hisF* gene. This is in agreement with previous data obtained for *E. coli* [39], highlighting that efficient recombination is linearly dependent on the length of the homologous sequences. Indeed, the extent of sequence homology influences the rate of the initial interaction and the stability of the heteroduplex structure [40]. No detectable differences were observed among the three different gene fragments based on the DNA length. These results suggested that the N-terminal region of *hisF* might be fundamental for the gene homologous recombination in the *E. coli* FB182 chromosome. An additional experiment was performed using the *E. coli* strain FB181 and the *hisIE* gene, demonstrating that this procedure is also efficient when using different genes. 

This work’s whole body of data demonstrated that the cloning-free genome editing procedure can be successfully applied to *E. coli* cells. The possibility of obtaining homologous recombination in *E. coli* starting from a circularized gene was demonstrated, and the simplicity of this procedure proposed by Döhlemann and coworkers [20] was confirmed. 

Moreover, since homologous recombination is an important evolutionary mechanism, our understanding of it needs to be deepened. One of the future perspectives might be the use of a synthetic *hisF* gene carrying synonymous mutations, homogeneously distributed along the gene; in this way, the obtained HisF protein would be identical to the wild-type one, but the *hisF* gene would be marked. Thus, after the CFGE experiment, the obtained recombinant colonies will be sequenced to evaluate the exact sites of recombination. In this way, it would be possible to observe if there are specific hyper-recombinogenic regions inside the *hisF* gene, or if the probability of recombination is independent of the gene region. Moreover, it would allow to better distinguish recombinant colonies from spontaneous chromosomal revertants.

Lastly, in addition to the possibility of using CFGE for generating site-specific deletions of a given DNA region as demonstrated by Dohlemann et al. [20], in the case of *S. meliloti*, *A. tumefaciens*, and *X. campestris*, the finding that two or more copies of the same amplified region can be integrated into the host chromosome might also open the way to evolutionary studies concerning the fate of in tandem duplicated genes. Indeed, it is known that gene duplication is one of the most important mechanisms driving the evolution of genes and genomes [41,42,43]. Once that a gene has duplicated, one of the two copies might accumulate mutations in such a way that the new gene (i.e., a paralog gene) acquires a metabolic ability different from the original one, thus increasing the metabolic potential of the cell.

In this context, the following experiments can be carried out in order to check the fate of duplicated genes: it can be imagined that, if the cell harboring two copies of the same gene is subjected to a selective pressure, one of the two copies will maintain the same function, whereas the other one might accumulate mutations of different types, or it might be lost over time. Thus, CFGE might also be used to explore the molecular rearrangements standing at the basis of genome evolution. 

## Figures and Tables

**Table 1:** Primers used in this work.

Primer Name	Primer Sequence (5′-3′)	Target Sequence	Amplicon
coli_hisF FW	ATGCTGGCAAAACGCATAA	*E. coli hisF* gene	*hisF*-777 bp *hisF2*-609 bp *hisF3*-408 bp *hisF4*-217 bp
coli_hisF_2 FW	GATGGCCGTGTGGTAGAT	*E. coli hisF* gene	
coli_hisF_3 FW	TGTATTGTGGTCGGTATTG	*E. coli hisF* gene	
coli_hisF_4 FW	TTACGACCTCGAACAACTG	*E. coli hisF* gene	
coli_hisF REV	TTAACATATCCTGATCTCCA	*E. coli hisF* gene	
coli_hisF_ext FW	GCGGCGTAATAGTTGGTCG	External to *E. coli hisF* gene	959 bp
coli_hisF_ext REV	TCTAAGGCTTCCGGGTTCAT	External to *E. coli hisF* gene	
coli_hisIE_ext FW	GCACCATGGAACACTTCCTC	External to *E. coli hisIE* gene	869 bp
coli_hisIE_ext REV	TACGCAATTACAACGCGAAG	External to *E. coli hisIE* gene	
M13 FW	GTAAAACGACGGCCAG	External to pGEM-T Easy MCS	variable
M13 REV	CAGGAAACAGCTATGAC	External to pGEM-T Easy MCS	

**Table 2:** Total number of His^+^ colonies obtained per experiment.

Experiment	Donor DNA			
	*hisF*	*hisF2*	*hisF3*	*hisF4*
	777 bp	609 bp	408 bp	217 bp
1	56	-	-	-
2	65	-	-	-
3	29	8	0	2
4	58	3	2	1
5	73	7	3	1
Total amount of His^+^ colonies	281	18	5	4
Mean	56.2	6	1.7	1.3

**Table 3:** Quality metrics of the genomic assemblies.

Colony ID	Experiment	Sample Group	N. Contigs	Largest Contig (bp)	Total Length (bp)	N50
FB182	-	-	1	4,637,980	4,637,980	4,637,980
40_E1	1	B	2	4,693,591	4,747,989	4,693,591
50_E2	2	B	1	4,666,291	4,666,291	4,666,291
55_E1	1	B	2	4,650,149	4,703,046	4,650,149
20_E2	2	B	2	4,645,202	4,745,589	4,645,202

**Table 4:** Summary about His^+^ colonies obtained after five CFGE experiments.

Experiment	Fragment		N. of His^+^ Colonies	N. of Characterized Colonies	Group A (1 *hisF* Copy)	Group B (2 or More *hisF* Copies)	Group C (Chromosomal Revertants)
	Name	Size (bp)					
1	*hisF*	777	56	20	3	17	0
2	*hisF*	777	65	20	4	15	1
3	*hisF*	777	29	20	3	16	1
	*hisF2*	609	8	8	5	3	0
	*hisF3*	408	0	0	0	0	0
	*hisF4*	217	2	2	1	0	1
4	*hisF*	777	58	20	6	14	0
	*hisF2*	609	3	3	0	2	1
	*hisF3*	408	2	2	0	1	1
	*hisF4*	217	1	1	0	0	1
5	*hisF*	777	73	20	2	18	0
	*hisF2*	609	7	7	1	6	0
	*hisF3*	408	3	3	0	0	3
	*hisF4*	217	1	1	0	0	1

## Data Availability

Publicly available data were used in this study. These data can be found here: https://www.ncbi.nlm.nih.gov/ (accessed on 4 January 2023) and https://alphafold.ebi.ac.uk/ (accessed on 4 January 2023). Accession numbers of the data used in this work are reported in the main text.

## Supplementary Materials

The following supporting information can be downloaded at: www.mdpi.com/article/10.3390/microorganisms11010215/s1, Table S1: ANOVA among the different groups defined on the basis of the donor DNA used for transformation; Table S2: ANOVA among the different groups defined on the basis of the different molecular rearrangement; Table S3: Transformation efficiency calculated for all the genes/gene fragments used in this study.
