# Supplementary material for: Application of Cloning-Free Genome Engineering to Escherichia coli
Source: Microorganisms. 2023 Jan 15;11(1):215. doi: 10.3390/microorganisms11010215 (PMC9866961; doi:10.3390/microorganisms11010215)
Supplement: Supplementary file 1 [file microorganisms-11-00215-s001.zip › microorganisms-2143849-supplementary.pdf]

Supplementary Material

Table S1. *p*-values of the analysis of variance (ANOVA) performed using Tukey's pairwise test, among the different groups defined on the basis of the donor DNA used for transformation. Significant *p*-values are highlighted in grey.

	hisF2	***hisF3***	***hisF4***
hisF	0.0003394	0.0001713	0.0001628
hisF2		0.957	0.9473
hisF3			1

Table S2. *p*-values of the analysis of variance (ANOVA) performed using Tukey's pairwise test, among the different groups defined on the basis of the different molecular rearrangement (group A: 1 *hisF* copy; group B: 2 or more *hisF* copies; group C: chromosomal revertants). Significant *p*-values are highlighted in grey.

	**B**	**C**
**A**	1,05E-08	0.005725
**B**		8,36E-10

Table S3. Transformation efficiency calculated for all the genes/gene fragments used in this study. Average number of transformants was calculated subtracting the number of chromosomal revertants (group C) from the number of obtained His+ colonies.

**Donor DNA**			**Average n. of transformants**	**Transformation efficiency (n. of transformants/μg of DNA)**
**Name**	**Length (bp)**	**Quantity (ng)**		
hisF	777	250	55.8	2.2×10^{2}
hisF2	609	200	5.7	2.9×10^{1}
hisF3	408	130	0.3	2.3×10^{0}
hisF4	217	70	0.3	4.3×10^{0}
hisIE	869	280	26	9.3×10^{1}
